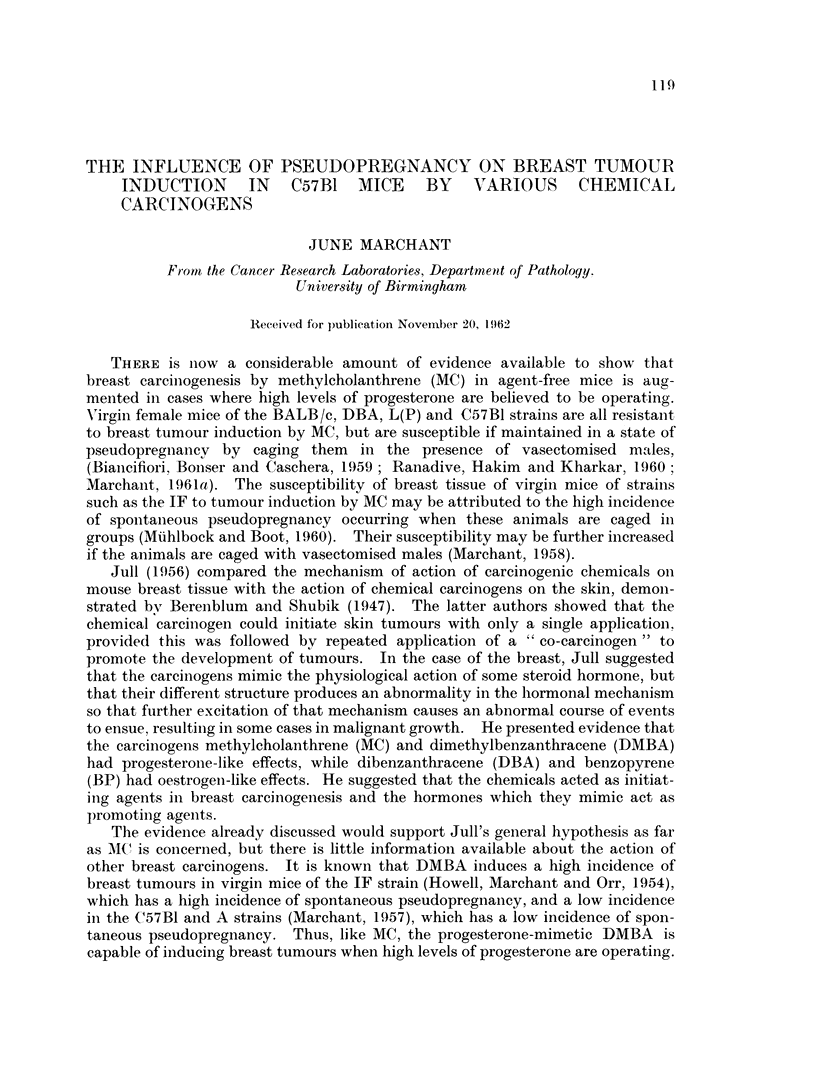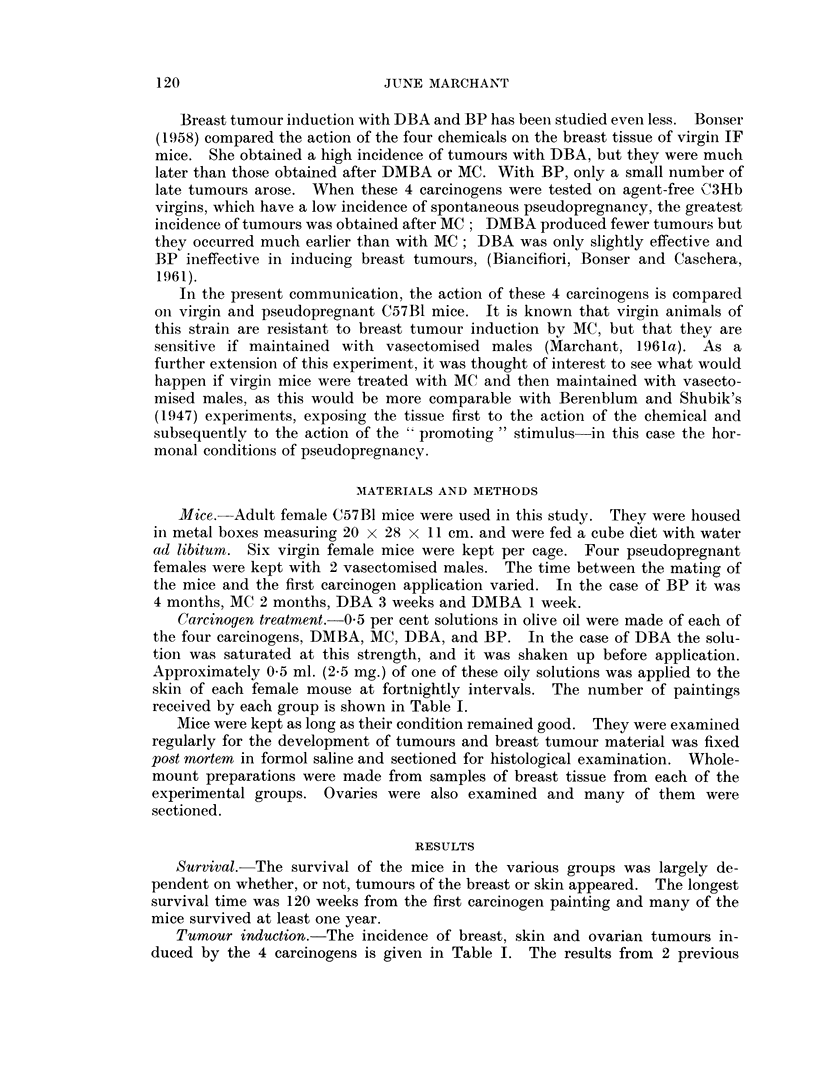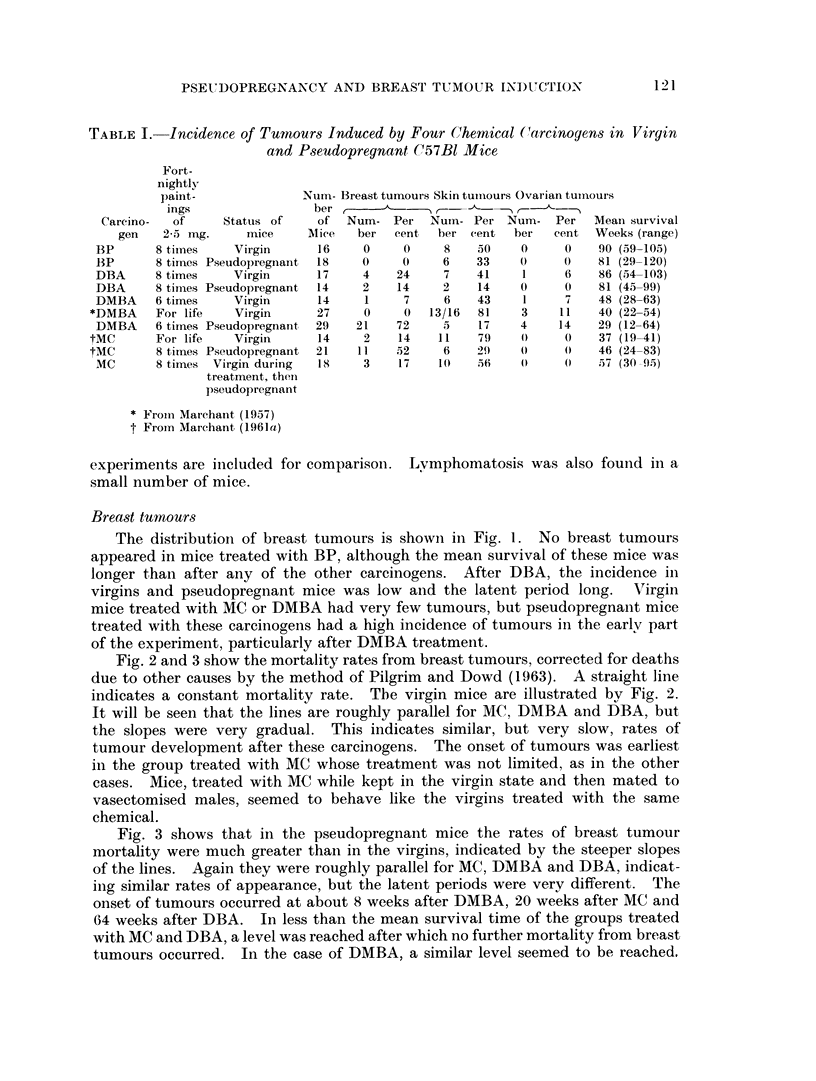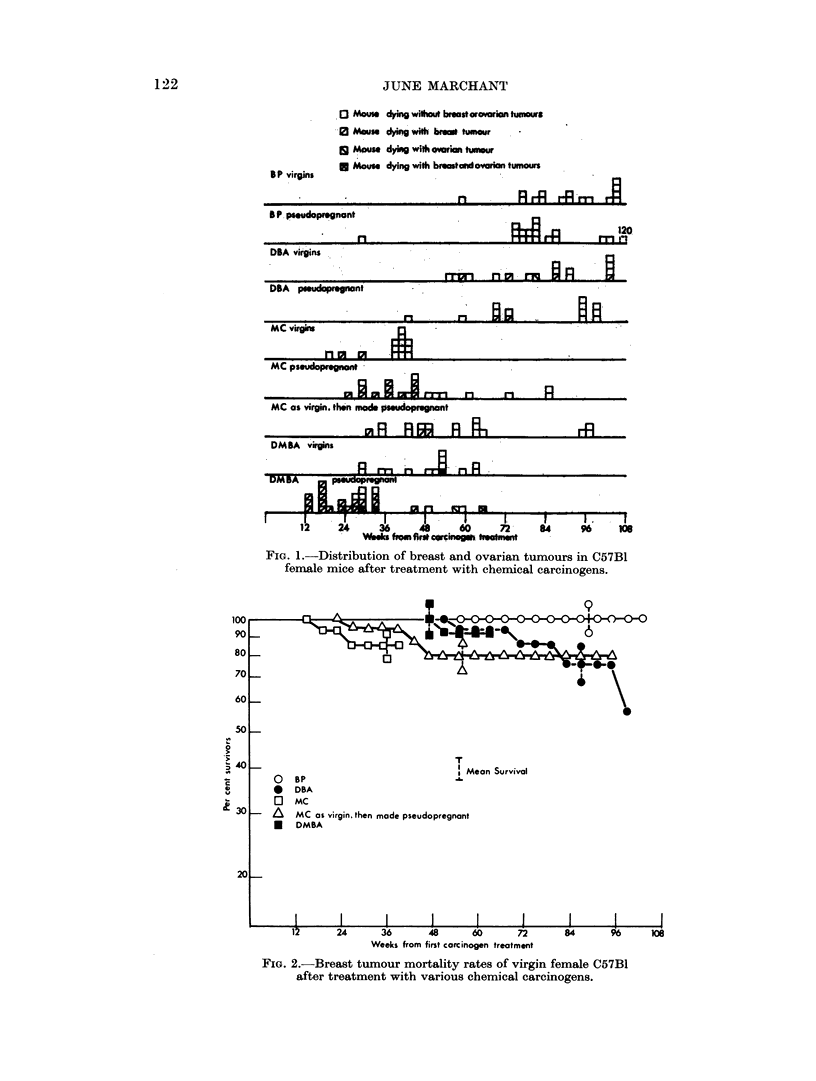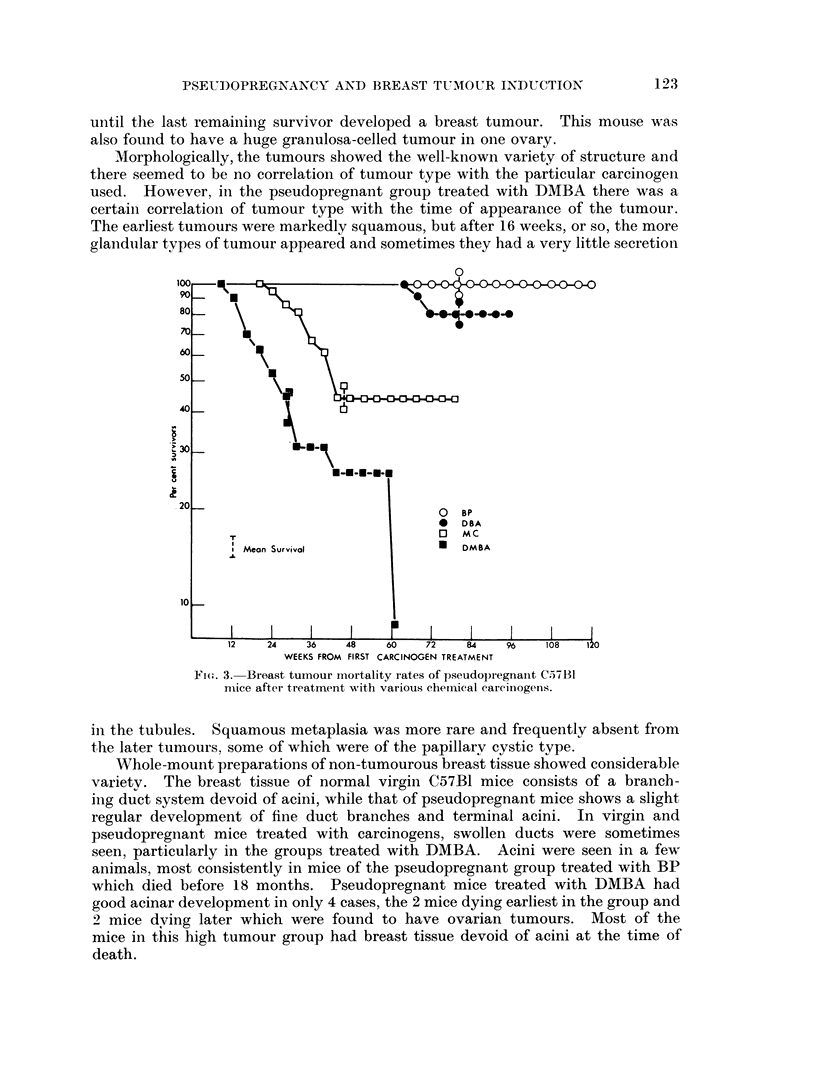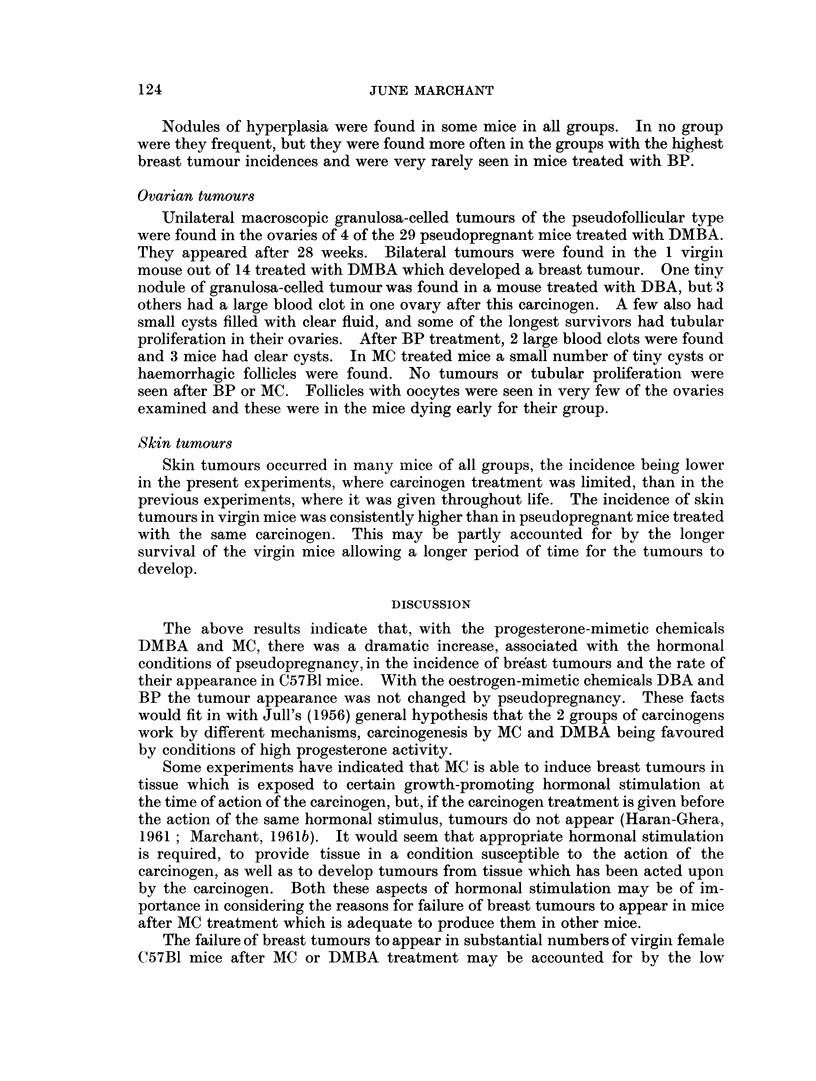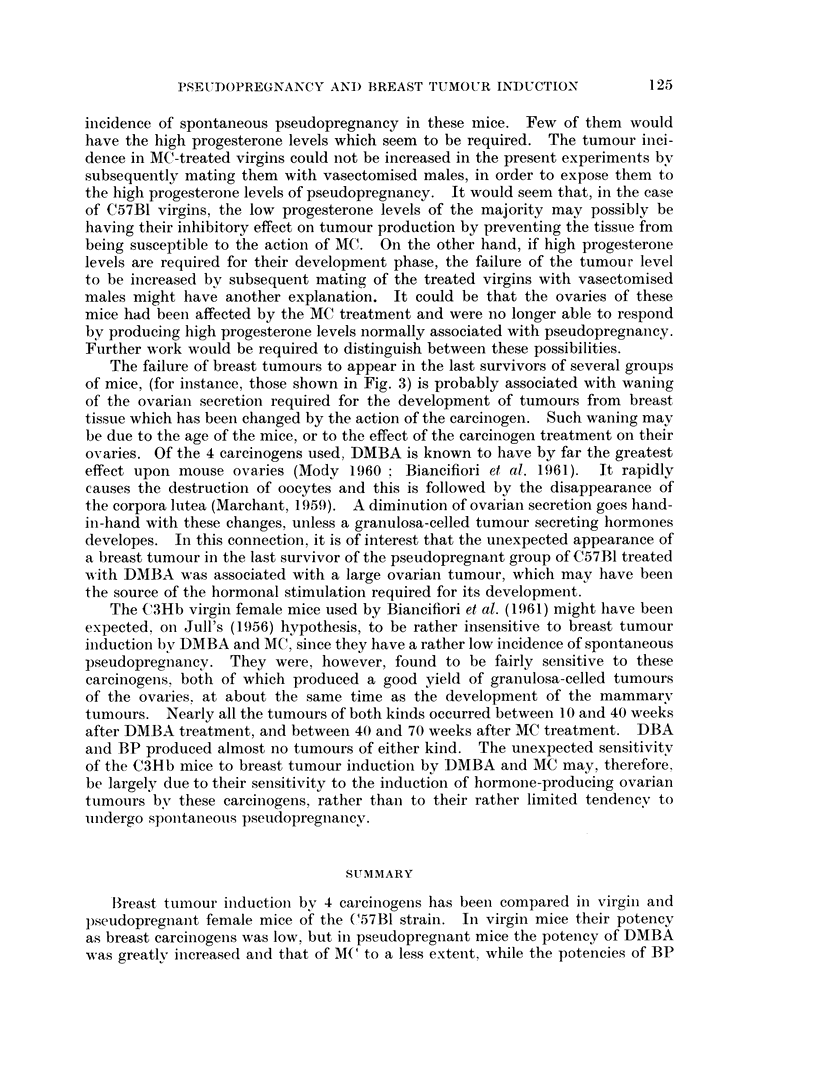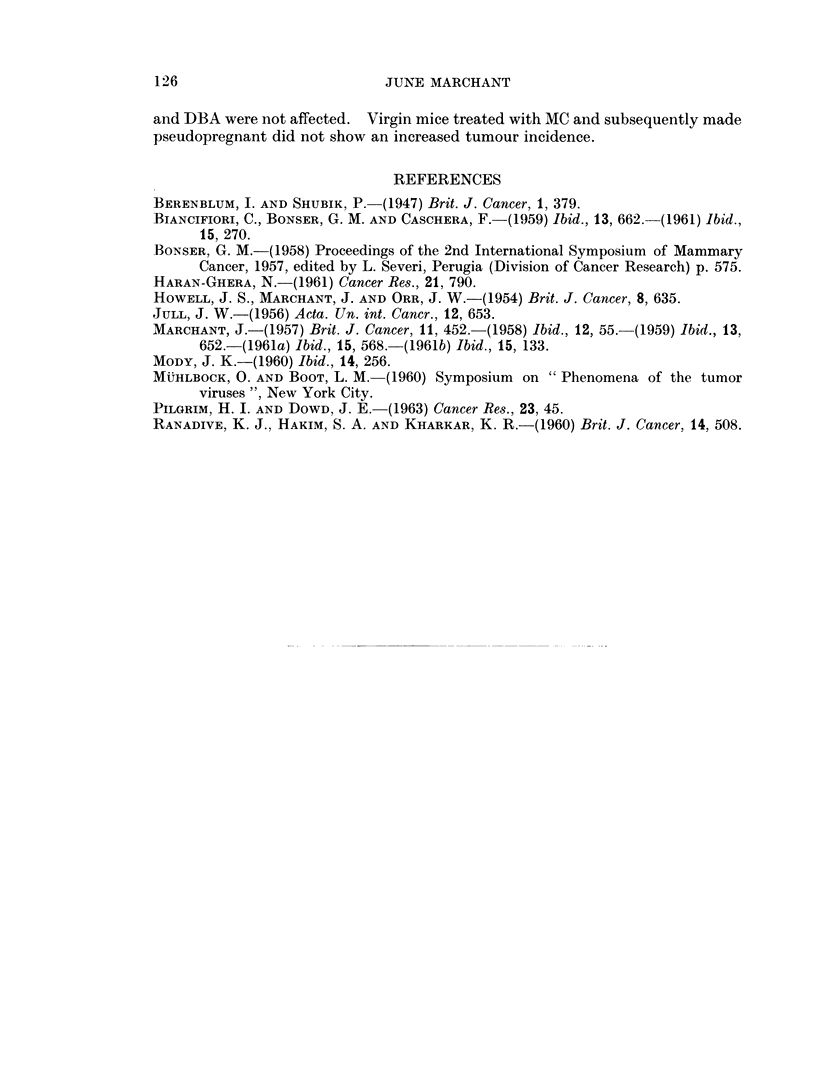# The Influence of Pseudopregnancy on Breast Tumour Induction in C57B1 Mice by Various Chemical Carcinogens

**DOI:** 10.1038/bjc.1963.18

**Published:** 1963-03

**Authors:** June Marchant


### THE INFLUENCE OF PSEUDOPREGNANCY ON BREAST TUMOUR INDUCTION IN C57Bl MICE BY VARIOUS CHEMICAL CARCINOGENS

JUNE MARCHANT

*From the Cancer Research Laboratories, Department of Pathology, University of Birmingham*

Received for publication November 20, 1962

THERE is now a considerable amount of evidence available to show that breast carcinogenesis by methylcholanthrene (MC) in agent-free mice is augmented in cases where high levels of progesterone are believed to be operating. Virgin female mice of the BALB/c, DBA, L(P) and C57Bl strains are all resistant to breast tumour induction by MC, but are susceptible if maintained in a state of pseudopregnancy by caging them in the presence of vasectomised males, (Biancifiori, Bonser and Caschera, 1959 ; Ranadive, Hakim and Kharkar, 1960 ; Marchant, 1961*a*). The susceptibility of breast tissue of virgin mice of strains such as the IF to tumour induction by MC may be attributed to the high incidence of spontaneous pseudopregnancy occurring when these animals are caged in groups (Mühlbock and Boot, 1960). Their susceptibility may be further increased if the animals are caged with vasectomised males (Marchant, 1958).

Jull (1956) compared the mechanism of action of carcinogenic chemicals on mouse breast tissue with the action of chemical carcinogens on the skin, demonstrated by Berenblum and Shubik (1947). The latter authors showed that the chemical carcinogen could initiate skin tumours with only a single application, provided this was followed by repeated application of a " co-carcinogen " to promote the development of tumours. In the case of the breast, Jull suggested that the carcinogens mimic the physiological action of some steroid hormone, but that their different structure produces an abnormality in the hormonal mechanism so that further excitation of that mechanism causes an abnormal course of events to ensue, resulting in some cases in malignant growth. He presented evidence that the carcinogens methylcholanthrene (MC) and dimethylbenzanthracene (DMBA) had progesterone-like effects, while dibenzanthracene (DBA) and benzopyrene (BP) had oestrogen-like effects. He suggested that the chemicals acted as initiating agents in breast carcinogenesis and the hormones which they mimic act as promoting agents.

The evidence already discussed would support Jull's general hypothesis as far as MC is concerned, but there is little information available about the action of other breast carcinogens. It is known that DMBA induces a high incidence of breast tumours in virgin mice of the IF strain (Howell, Marchant and Orr, 1954), which has a high incidence of spontaneous pseudopregnancy, and a low incidence in the C57Bl and A strains (Marchant, 1957), which has a low incidence of spontaneous pseudopregnancy. Thus, like MC, the progesterone-mimetic DMBA is capable of inducing breast tumours when high levels of progesterone are operating.

Breast tumour induction with DBA and BP has been studied even less. Bonser (1958) compared the action of the four chemicals on the breast tissue of virgin IF mice. She obtained a high incidence of tumours with DBA, but they were much later than those obtained after DMBA or MC. With BP, only a small number of late tumours arose. When these 4 carcinogens were tested on agent-free C3Hb virgins, which have a low incidence of spontaneous pseudopregnancy, the greatest incidence of tumours was obtained after MC ; DMBA produced fewer tumours but they occurred much earlier than with MC ; DBA was only slightly effective and BP ineffective in inducing breast tumours, (Biancifiori, Bonser and Caschera, 1961).

In the present communication, the action of these 4 carcinogens is compared on virgin and pseudopregnant C57Bl mice. It is known that virgin animals of this strain are resistant to breast tumour induction by MC, but that they are sensitive if maintained with vasectomised males (Marchant, 1961*a*). As a further extension of this experiment, it was thought of interest to see what would happen if virgin mice were treated with MC and then maintained with vasectomised males, as this would be more comparable with Berenblum and Shubik's (1947) experiments, exposing the tissue first to the action of the chemical and subsequently to the action of the " promoting " stimulus—in this case the hormonal conditions of pseudopregnancy.

### MATERIALS AND METHODS

*Mice.*—Adult female C57Bl mice were used in this study. They were housed in metal boxes measuring 20 × 28 × 11 cm. and were fed a cube diet with water *ad libitum.* Six virgin female mice were kept per cage. Four pseudopregnant females were kept with 2 vasectomised males. The time between the mating of the mice and the first carcinogen application varied. In the case of BP it was 4 months, MC 2 months, DBA 3 weeks and DMBA 1 week.

*Carcinogen treatment.*—0·5 per cent solutions in olive oil were made of each of the four carcinogens, DMBA, MC, DBA, and BP. In the case of DBA the solution was saturated at this strength, and it was shaken up before application. Approximately 0·5 ml. (2·5 mg.) of one of these oily solutions was applied to the skin of each female mouse at fortnightly intervals. The number of paintings received by each group is shown in Table I.

Mice were kept as long as their condition remained good. They were examined regularly for the development of tumours and breast tumour material was fixed *post mortem* in formol saline and sectioned for histological examination. Whole-mount preparations were made from samples of breast tissue from each of the experimental groups. Ovaries were also examined and many of them were sectioned.

### RESULTS

*Survival.*—The survival of the mice in the various groups was largely dependent on whether, or not, tumours of the breast or skin appeared. The longest survival time was 120 weeks from the first carcinogen painting and many of the mice survived at least one year.

*Tumour induction.*—The incidence of breast, skin and ovarian tumours induced by the 4 carcinogens is given in Table I. The results from 2 previous

TABLE I.—*Incidence of Tumours Induced by Four Chemical Carcinogens in Virgin and Pseudopregnant C57Bl Mice*

| Carcinogen | Fortnightly paintings of 2·5 mg. | Status of mice | Number of Mice | Breast tumours Number | Breast tumours Per cent | Skin tumours Number | Skin tumours Per cent | Ovarian tumours Number | Ovarian tumours Per cent | Mean survival Weeks (range) |
|---|---|---|---|---|---|---|---|---|---|---|
| BP | 8 times | Virgin | 16 | 0 | 0 | 8 | 50 | 0 | 0 | 90 (59–105) |
| BP | 8 times | Pseudopregnant | 18 | 0 | 0 | 6 | 33 | 0 | 0 | 81 (29–120) |
| DBA | 8 times | Virgin | 17 | 4 | 24 | 7 | 41 | 1 | 6 | 86 (54–103) |
| DBA | 8 times | Pseudopregnant | 14 | 2 | 14 | 2 | 14 | 0 | 0 | 81 (45–99) |
| DMBA | 6 times | Virgin | 14 | 1 | 7 | 6 | 43 | 1 | 7 | 48 (28–63) |
| *DMBA | For life | Virgin | 27 | 0 | 0 | 13/16 | 81 | 3 | 11 | 40 (22–54) |
| DMBA | 6 times | Pseudopregnant | 29 | 21 | 72 | 5 | 17 | 4 | 14 | 29 (12–64) |
| †MC | For life | Virgin | 14 | 2 | 14 | 11 | 79 | 0 | 0 | 37 (19–41) |
| †MC | 8 times | Pseudopregnant | 21 | 11 | 52 | 6 | 29 | 0 | 0 | 46 (24–83) |
| MC | 8 times | Virgin during treatment, then pseudopregnant | 18 | 3 | 17 | 10 | 56 | 0 | 0 | 57 (30–95) |

* From Marchant (1957)

† From Marchant (1961*a*)

experiments are included for comparison. Lymphomatosis was also found in a small number of mice.

*Breast tumours*

The distribution of breast tumours is shown in Fig. 1. No breast tumours appeared in mice treated with BP, although the mean survival of these mice was longer than after any of the other carcinogens. After DBA, the incidence in virgins and pseudopregnant mice was low and the latent period long. Virgin mice treated with MC or DMBA had very few tumours, but pseudopregnant mice treated with these carcinogens had a high incidence of tumours in the early part of the experiment, particularly after DMBA treatment.

Fig. 2 and 3 show the mortality rates from breast tumours, corrected for deaths due to other causes by the method of Pilgrim and Dowd (1963). A straight line indicates a constant mortality rate. The virgin mice are illustrated by Fig. 2. It will be seen that the lines are roughly parallel for MC, DMBA and DBA, but the slopes were very gradual. This indicates similar, but very slow, rates of tumour development after these carcinogens. The onset of tumours was earliest in the group treated with MC whose treatment was not limited, as in the other cases. Mice, treated with MC while kept in the virgin state and then mated to vasectomised males, seemed to behave like the virgins treated with the same chemical.

Fig. 3 shows that in the pseudopregnant mice the rates of breast tumour mortality were much greater than in the virgins, indicated by the steeper slopes of the lines. Again they were roughly parallel for MC, DMBA and DBA, indicating similar rates of appearance, but the latent periods were very different. The onset of tumours occurred at about 8 weeks after DMBA, 20 weeks after MC and 64 weeks after DBA. In less than the mean survival time of the groups treated with MC and DBA, a level was reached after which no further mortality from breast tumours occurred. In the case of DMBA, a similar level seemed to be reached.

**Fig. 1.—Distribution of breast and ovarian tumours in C57Bl female mice after treatment with chemical carcinogens.**

**Fig. 2.—Breast tumour mortality rates of virgin female C57Bl after treatment with various chemical carcinogens.**

until the last remaining survivor developed a breast tumour. This mouse was also found to have a huge granulosa-celled tumour in one ovary.

Morphologically, the tumours showed the well-known variety of structure and there seemed to be no correlation of tumour type with the particular carcinogen used. However, in the pseudopregnant group treated with DMBA there was a certain correlation of tumour type with the time of appearance of the tumour. The earliest tumours were markedly squamous, but after 16 weeks, or so, the more glandular types of tumour appeared and sometimes they had a very little secretion

FIG. 3.—Breast tumour mortality rates of pseudopregnant C57Bl mice after treatment with various chemical carcinogens.

in the tubules. Squamous metaplasia was more rare and frequently absent from the later tumours, some of which were of the papillary cystic type.

Whole-mount preparations of non-tumourous breast tissue showed considerable variety. The breast tissue of normal virgin C57Bl mice consists of a branching duct system devoid of acini, while that of pseudopregnant mice shows a slight regular development of fine duct branches and terminal acini. In virgin and pseudopregnant mice treated with carcinogens, swollen ducts were sometimes seen, particularly in the groups treated with DMBA. Acini were seen in a few animals, most consistently in mice of the pseudopregnant group treated with BP which died before 18 months. Pseudopregnant mice treated with DMBA had good acinar development in only 4 cases, the 2 mice dying earliest in the group and 2 mice dying later which were found to have ovarian tumours. Most of the mice in this high tumour group had breast tissue devoid of acini at the time of death.

Nodules of hyperplasia were found in some mice in all groups. In no group were they frequent, but they were found more often in the groups with the highest breast tumour incidences and were very rarely seen in mice treated with BP.

*Ovarian tumours*

Unilateral macroscopic granulosa-celled tumours of the pseudofollicular type were found in the ovaries of 4 of the 29 pseudopregnant mice treated with DMBA. They appeared after 28 weeks. Bilateral tumours were found in the 1 virgin mouse out of 14 treated with DMBA which developed a breast tumour. One tiny nodule of granulosa-celled tumour was found in a mouse treated with DBA, but 3 others had a large blood clot in one ovary after this carcinogen. A few also had small cysts filled with clear fluid, and some of the longest survivors had tubular proliferation in their ovaries. After BP treatment, 2 large blood clots were found and 3 mice had clear cysts. In MC treated mice a small number of tiny cysts or haemorrhagic follicles were found. No tumours or tubular proliferation were seen after BP or MC. Follicles with oocytes were seen in very few of the ovaries examined and these were in the mice dying early for their group.

*Skin tumours*

Skin tumours occurred in many mice of all groups, the incidence being lower in the present experiments, where carcinogen treatment was limited, than in the previous experiments, where it was given throughout life. The incidence of skin tumours in virgin mice was consistently higher than in pseudopregnant mice treated with the same carcinogen. This may be partly accounted for by the longer survival of the virgin mice allowing a longer period of time for the tumours to develop.

### DISCUSSION

The above results indicate that, with the progesterone-mimetic chemicals DMBA and MC, there was a dramatic increase, associated with the hormonal conditions of pseudopregnancy, in the incidence of breast tumours and the rate of their appearance in C57Bl mice. With the oestrogen-mimetic chemicals DBA and BP the tumour appearance was not changed by pseudopregnancy. These facts would fit in with Jull's (1956) general hypothesis that the 2 groups of carcinogens work by different mechanisms, carcinogenesis by MC and DMBA being favoured by conditions of high progesterone activity.

Some experiments have indicated that MC is able to induce breast tumours in tissue which is exposed to certain growth-promoting hormonal stimulation at the time of action of the carcinogen, but, if the carcinogen treatment is given before the action of the same hormonal stimulus, tumours do not appear (Haran-Ghera, 1961 ; Marchant, 1961*b*). It would seem that appropriate hormonal stimulation is required, to provide tissue in a condition susceptible to the action of the carcinogen, as well as to develop tumours from tissue which has been acted upon by the carcinogen. Both these aspects of hormonal stimulation may be of importance in considering the reasons for failure of breast tumours to appear in mice after MC treatment which is adequate to produce them in other mice.

The failure of breast tumours to appear in substantial numbers of virgin female C57Bl mice after MC or DMBA treatment may be accounted for by the low incidence of spontaneous pseudopregnancy in these mice. Few of them would have the high progesterone levels which seem to be required. The tumour incidence in MC-treated virgins could not be increased in the present experiments by subsequently mating them with vasectomised males, in order to expose them to the high progesterone levels of pseudopregnancy. It would seem that, in the case of C57Bl virgins, the low progesterone levels of the majority may possibly be having their inhibitory effect on tumour production by preventing the tissue from being susceptible to the action of MC. On the other hand, if high progesterone levels are required for their development phase, the failure of the tumour level to be increased by subsequent mating of the treated virgins with vasectomised males might have another explanation. It could be that the ovaries of these mice had been affected by the MC treatment and were no longer able to respond by producing high progesterone levels normally associated with pseudopregnancy. Further work would be required to distinguish between these possibilities.

The failure of breast tumours to appear in the last survivors of several groups of mice, (for instance, those shown in Fig. 3) is probably associated with waning of the ovarian secretion required for the development of tumours from breast tissue which has been changed by the action of the carcinogen. Such waning may be due to the age of the mice, or to the effect of the carcinogen treatment on their ovaries. Of the 4 carcinogens used, DMBA is known to have by far the greatest effect upon mouse ovaries (Mody 1960 ; Biancifiori *et al.* 1961). It rapidly causes the destruction of oocytes and this is followed by the disappearance of the corpora lutea (Marchant, 1959). A diminution of ovarian secretion goes hand-in-hand with these changes, unless a granulosa-celled tumour secreting hormones developes. In this connection, it is of interest that the unexpected appearance of a breast tumour in the last survivor of the pseudopregnant group of C57Bl treated with DMBA was associated with a large ovarian tumour, which may have been the source of the hormonal stimulation required for its development.

The C3Hb virgin female mice used by Biancifiori *et al.* (1961) might have been expected, on Jull's (1956) hypothesis, to be rather insensitive to breast tumour induction by DMBA and MC, since they have a rather low incidence of spontaneous pseudopregnancy. They were, however, found to be fairly sensitive to these carcinogens, both of which produced a good yield of granulosa-celled tumours of the ovaries, at about the same time as the development of the mammary tumours. Nearly all the tumours of both kinds occurred between 10 and 40 weeks after DMBA treatment, and between 40 and 70 weeks after MC treatment. DBA and BP produced almost no tumours of either kind. The unexpected sensitivity of the C3Hb mice to breast tumour induction by DMBA and MC may, therefore, be largely due to their sensitivity to the induction of hormone-producing ovarian tumours by these carcinogens, rather than to their rather limited tendency to undergo spontaneous pseudopregnancy.

SUMMARY

Breast tumour induction by 4 carcinogens has been compared in virgin and pseudopregnant female mice of the C57Bl strain. In virgin mice their potency as breast carcinogens was low, but in pseudopregnant mice the potency of DMBA was greatly increased and that of MC to a less extent, while the potencies of BP

and DBA were not affected. Virgin mice treated with MC and subsequently made pseudopregnant did not show an increased tumour incidence.

### REFERENCES

BERENBLUM, I. AND SHUBIK, P.—(1947) *Brit. J. Cancer*, **1**, 379.

BIANCIFIORI, C., BONSER, G. M. AND CASCHERA, F.—(1959) *Ibid.*, **13**, 662.—(1961) *Ibid.*, **15**, 270.

BONSER, G. M.—(1958) Proceedings of the 2nd International Symposium of Mammary Cancer, 1957, edited by L. Severi, Perugia (Division of Cancer Research) p. 575.

HARAN-GHERA, N.—(1961) *Cancer Res.*, **21**, 790.

HOWELL, J. S., MARCHANT, J. AND ORR, J. W.—(1954) *Brit. J. Cancer*, **8**, 635.

JULL, J. W.—(1956) *Acta. Un. int. Cancr.*, **12**, 653.

MARCHANT, J.—(1957) *Brit. J. Cancer*, **11**, 452.—(1958) *Ibid.*, **12**, 55.—(1959) *Ibid.*, **13**, 652.—(1961*a*) *Ibid.*, **15**, 568.—(1961*b*) *Ibid.*, **15**, 133.

MODY, J. K.—(1960) *Ibid.*, **14**, 256.

MÜHLBOCK, O. AND BOOT, L. M.—(1960) Symposium on " Phenomena of the tumor viruses ", New York City.

PILGRIM, H. I. AND DOWD, J. E.—(1963) *Cancer Res.*, **23**, 45.

RANADIVE, K. J., HAKIM, S. A. AND KHARKAR, K. R.—(1960) *Brit. J. Cancer*, **14**, 508.